# Recurrent Evisceration After Cystoprostatectomy in a Multimorbid Patient: A Case Report

**DOI:** 10.7759/cureus.92036

**Published:** 2025-09-11

**Authors:** Jan Stępka, Maciej Konopka, Tomasz Milecki, Konstanty Drogowski, Wojciech Cieślikowski, Tomasz Deja

**Affiliations:** 1 Department of Urology and Oncological Urology, University Clinical Hospital, Poznan, POL; 2 Department of Urology, Ministry of Internal Affairs Hospital, Poznan, POL; 3 Department of Urology, Poznań University of Medical Sciences, Poznan, POL

**Keywords:** abdominal wall reconstruction, bladder cancer surgery, evisceration, multimorbid patient, musculocutaneous flap, negative pressure wound therapy, postoperative complications, radical cystoprostatectomy, split-thickness skin graft, wound dehiscence

## Abstract

We report a rare case of recurrent evisceration following radical cystoprostatectomy with Bricker urinary diversion in a 71-year-old patient with type 2 diabetes mellitus, chronic obstructive pulmonary disease, hypertension, and peripheral arterial disease. Despite an initially uneventful recovery, the patient presented with full-thickness wound dehiscence and evisceration just eight days after discharge. His recovery was further complicated by comorbidities and poor adherence to postoperative activity restrictions. After several abdominal wall re-explorations and debridements, combined with negative pressure wound therapy (NPWT), definitive reconstruction was achieved using a pedicled musculocutaneous flap, followed by split-thickness skin grafting. At 10-month follow-up, the patient achieved full wound healing with satisfactory functional and cosmetic outcomes. This case highlights the importance of risk stratification, early use of advanced wound management techniques, and multidisciplinary care in complex surgical patients.

## Introduction

Radical cystoprostatectomy remains the gold standard treatment for muscle-invasive bladder cancer [1]. Although surgical techniques and perioperative care have significantly improved in recent years, postoperative complications such as wound dehiscence and evisceration continue to present clinical challenges, particularly in comorbid patients undergoing extensive procedures. Studies have reported that abdominal wall complications, including impaired wound healing, occur in approximately 1.2% of patients following planned laparotomy [2]. Among these, evisceration is considered a rare but severe complication, accounting for less than 0.5% of cases after laparotomy [3-6]. To our knowledge, no previously published case has documented recurrent episodes of evisceration following radical cystoprostatectomy. In light of this, we present a unique case of a patient who experienced multiple episodes of postoperative evisceration requiring repeated reconstructive surgical intervention. This case highlights the importance of thorough preoperative risk assessment in complex surgical candidates, as well as the successful management of the condition through staged abdominal wall reconstruction using a pedicled musculocutaneous flap followed by split-thickness skin grafting.

## Case presentation

A 71-year-old man was admitted for elective radical cystoprostatectomy due to a diagnosis of high-grade, muscle-invasive urothelial carcinoma (pathological stage T2, pT2) confirmed on transurethral resection of bladder tumor (TURBT) in January 2023. A computed tomography (CT) scan in June 2023 showed no signs of metastatic disease. Before surgery, the patient received neoadjuvant immunotherapy with enfortumab vedotin and pembrolizumab.

His medical history was significant for type 2 diabetes mellitus, arterial hypertension, chronic obstructive pulmonary disease (COPD), ischemic stroke (2012), peripheral arterial disease with prior endovascular treatment, degenerative lumbar spine disease, and heavy nicotine use (15 cigarettes/day for 50 years). He was functionally independent but presented with diminished mobility and chronic fatigue. His body mass index (BMI) was 25.9 kg/m², hemoglobin (Hb) was 13.4 g/dL, and his American Society of Anesthesiologists (ASA) score was 4. Notably, his surgical history included two prior TURBTs and a right-sided inguinal hernia repair with mesh placement.

On November 2, 2023, the patient underwent radical cystoprostatectomy with urinary diversion using an ileal conduit (Bricker technique). The procedure included en bloc resection of the bladder, prostate, and seminal vesicles, as well as bilateral extended pelvic lymph node dissection and drain insertion. The surgery was completed without immediate intraoperative complications. The patient was extubated postoperatively and transferred from the intensive care unit to the urology ward. His early postoperative course progressed without incident, and he was discharged on postoperative day six with standard instructions regarding wound care, stoma management, and activity restrictions.

**Figure 1:**
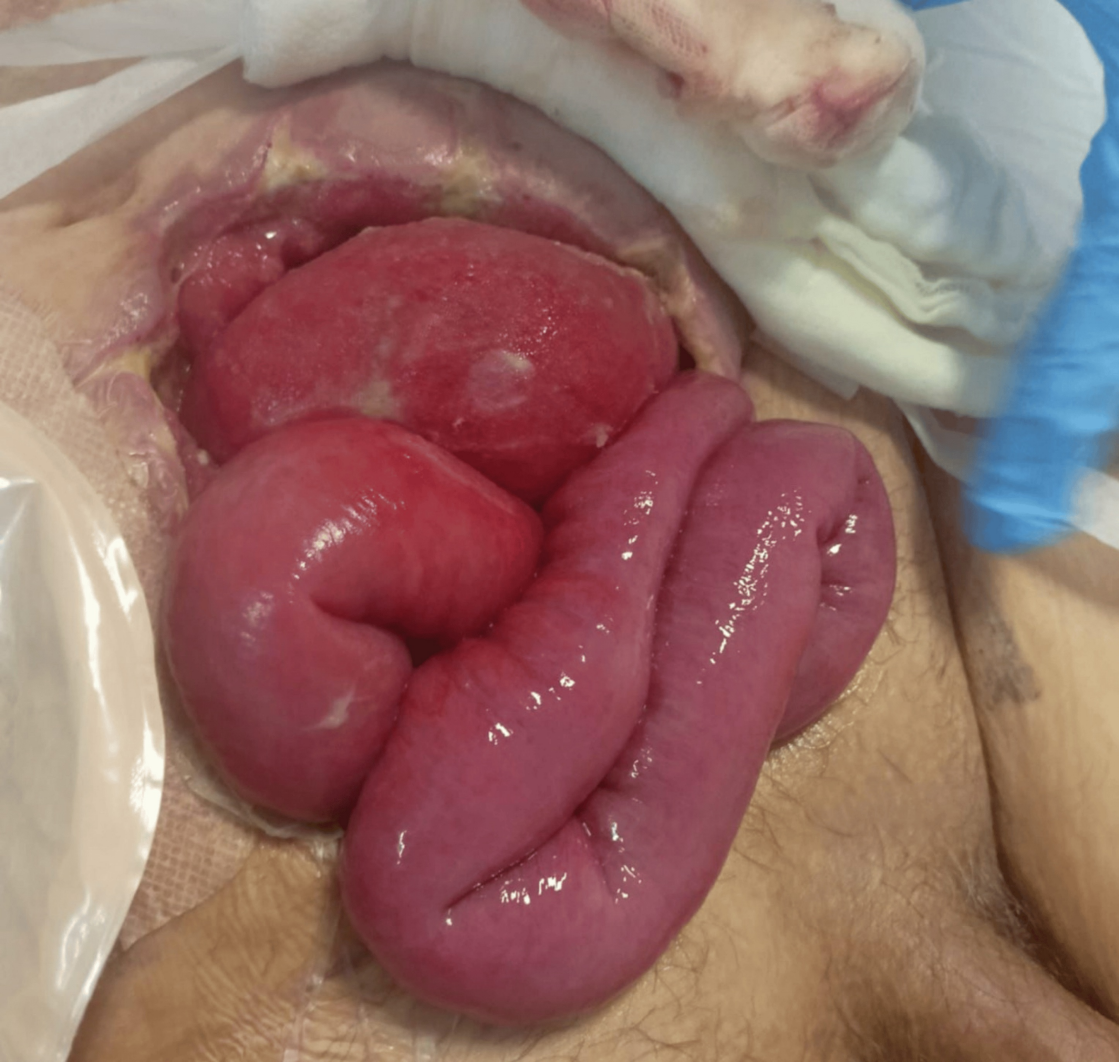
Evisceration through abdominal wall defect. Clinical photograph showing full-thickness wound dehiscence with protrusion of abdominal contents through the defect.

**Figure 2:**
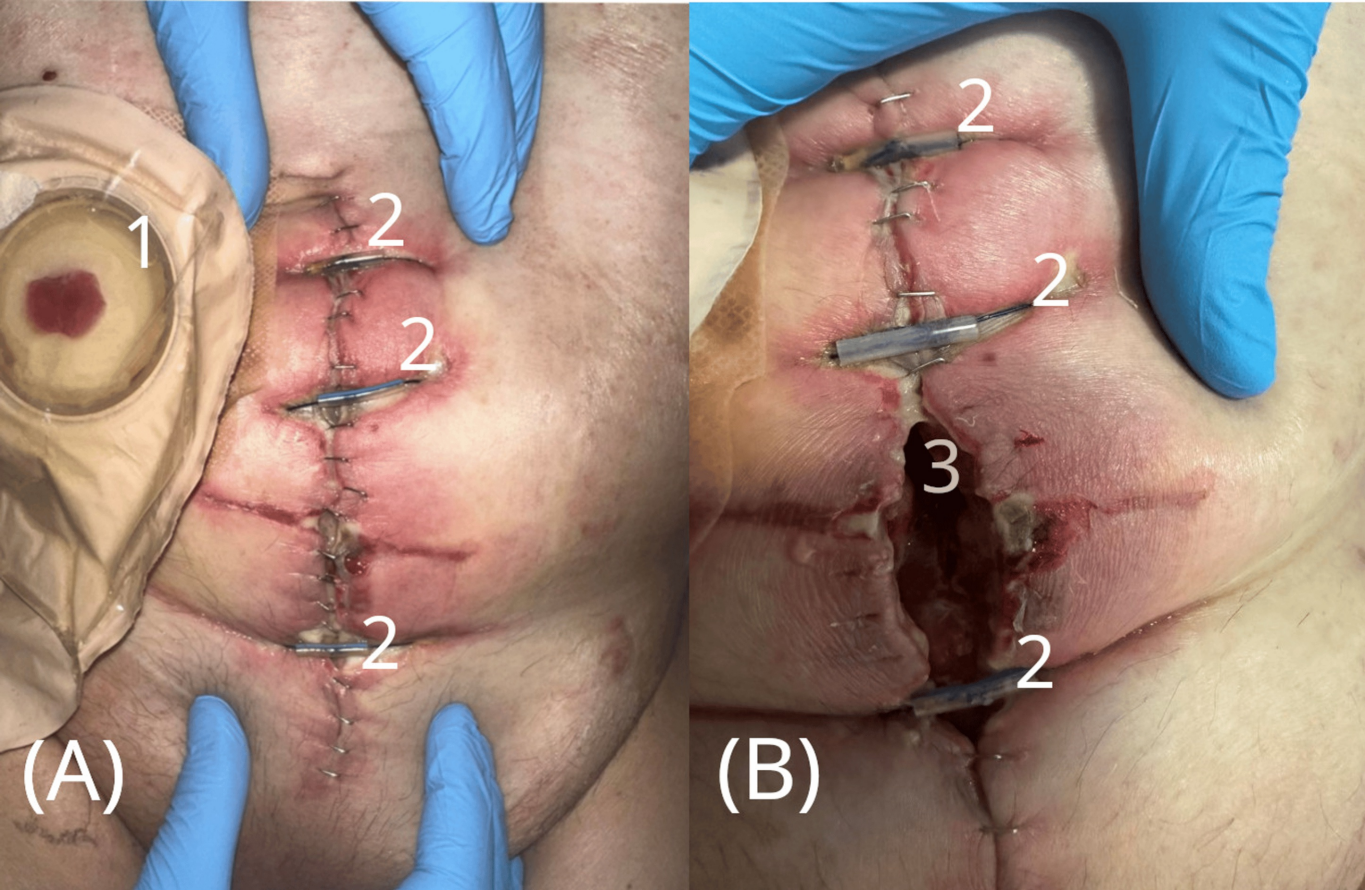
Postoperative course after the first evisceration repair. (A) Immediate postoperative appearance following emergency closure with retention sutures. (B) Wound dehiscence was observed several days later, resulting in a new defect. 1 - Ileal conduit stoma with urostomy bag 2 - Retention (anti-evisceration) sutures 3 - New abdominal wall defect due to wound dehiscence

**Figure 3:**
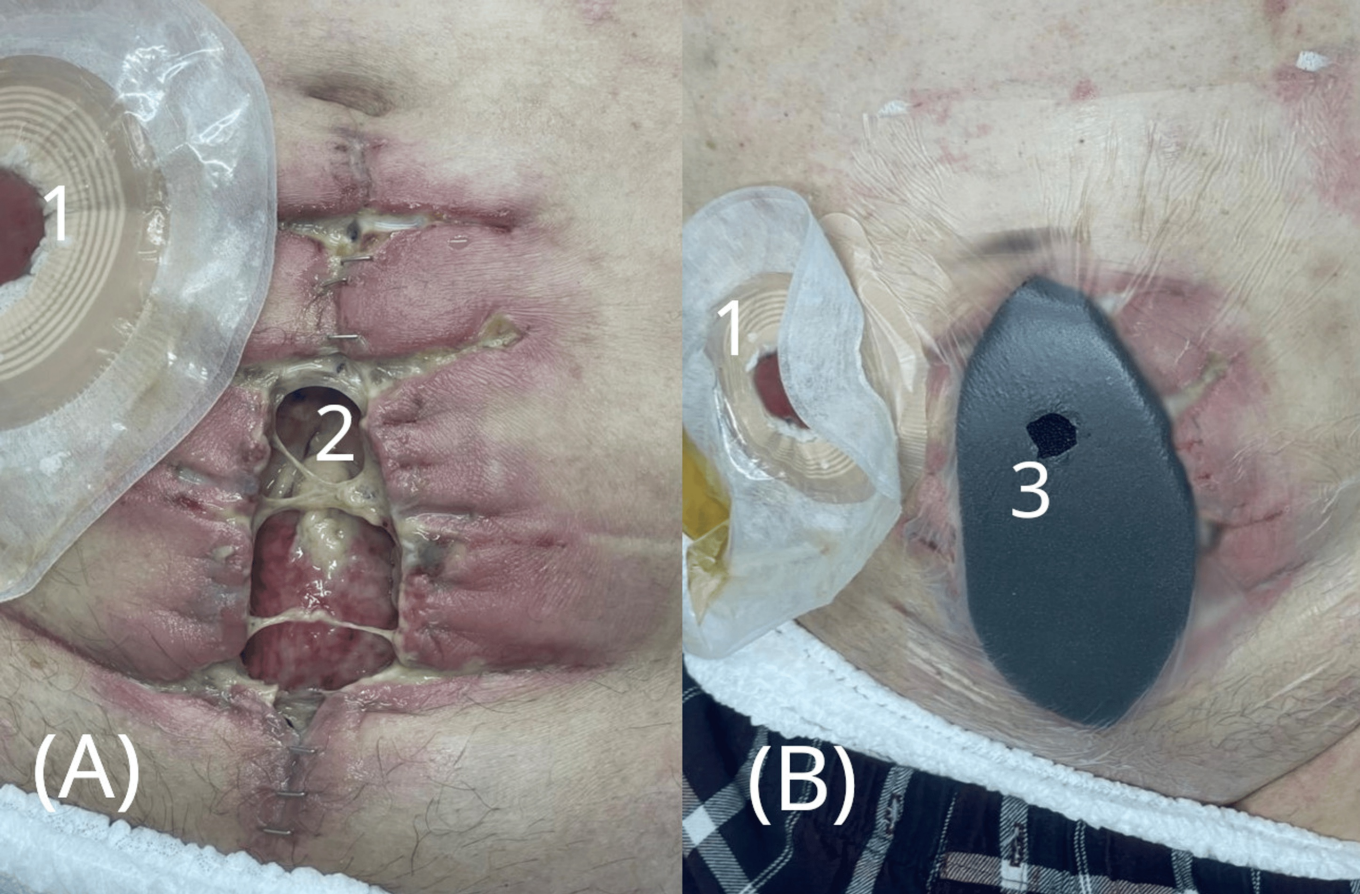
Wound condition before and after initiation of negative pressure therapy. (A) Clinical image showing wound deterioration after repeat dehiscence, with visible purulent discharge. (B) Application of negative pressure wound therapy (NPWT). 1 - Ileal conduit stoma with urostomy bag 2 - Reopened wound with signs of infection and purulent exudate 3 - NPWT dressing in place

On November 17, 2023, eight days after discharge, the patient presented with complete wound dehiscence and evisceration of abdominal contents (Figure 1). The event reportedly occurred while the patient was mopping the kitchen floor. Emergency surgical intervention was performed with abdominal wall closure and placement of retention sutures (Figure 2A). Despite this intervention, the wound reopened (Figure 2B). Due to progressive deterioration of the wound (Figure 3A), a consultation with the plastic surgery department was undertaken. Negative pressure wound therapy (NPWT) was initiated, and further reconstructive steps were planned (Figure 3B).

**Figure 4:**
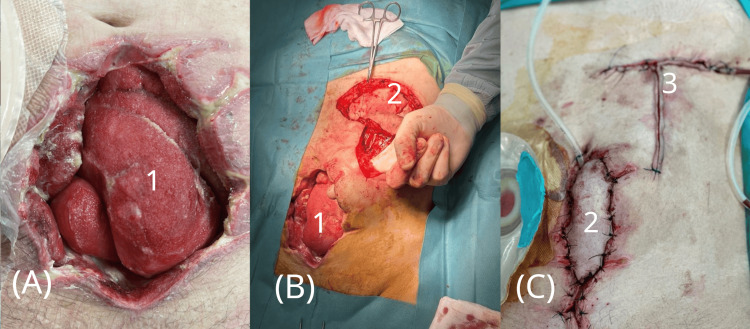
Abdominal wall reconstruction with a pedicled musculocutaneous flap. (A) Healed but persistent wound after negative pressure wound therapy (NPWT), before reconstructive surgery. (B) Intraoperative view showing mobilization of the pedicled musculocutaneous flap. (C) The transposed flap was sutured into the abdominal wall defect, and the donor site was closed. 1 - Granulated wound following NPWT 2 - Mobilized pedicled musculocutaneous flap 3 - Sutured donor site following flap harvest

On December 2, 2023, as the wound bed began to granulate, the medical team decided to perform abdominal wall reconstruction to address the defect (Figure 4A). To restore continuity in the medial portion of the upper left abdominal quadrant, a pedicled musculocutaneous flap based on the inferior epigastric artery was prepared. The flap was harvested and transposed to the site of the defect (Figure 4B). The patency of the epigastric artery was confirmed, the flap was sutured in place, the donor site was closed, and a drain was inserted (Figure 4C). This complex reconstructive procedure was conducted in collaboration with a plastic surgeon. The patient remained under close observation during recovery.

**Figure 5:**
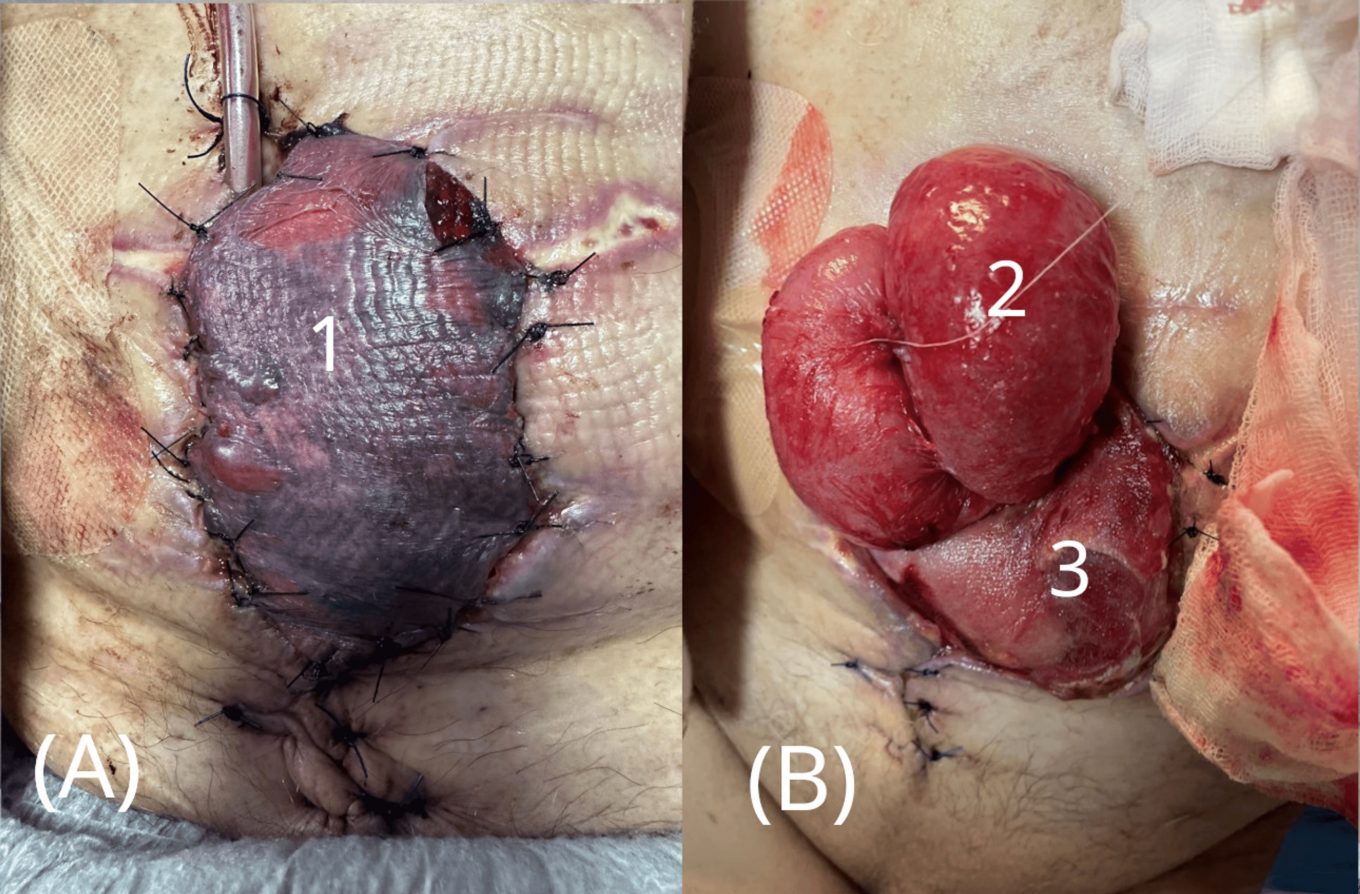
Flap deterioration and recurrent evisceration. (A) Clinical image showing progressive necrosis of the cutaneous portion of the transposed flap, likely due to venous insufficiency. (B) Recurrent evisceration at the junction between the original abdominal wall and the transplanted flap. 1 - Cutaneous flap necrosis secondary to venous insufficiency 2 - Eviscerated abdominal contents near the flap site 3 - Transplanted musculocutaneous flap

In the days that followed, due to venous insufficiency, partial necrosis of the cutaneous portion of the flap occurred (Figure 5A). On December 11, 2025, the patient experienced another episode of wound dehiscence and evisceration. This time, the defect occurred at the junction between the original abdominal wall and the transposed flap (Figure 5B), exposing both the flap and intra-abdominal contents.

**Figure 6:**
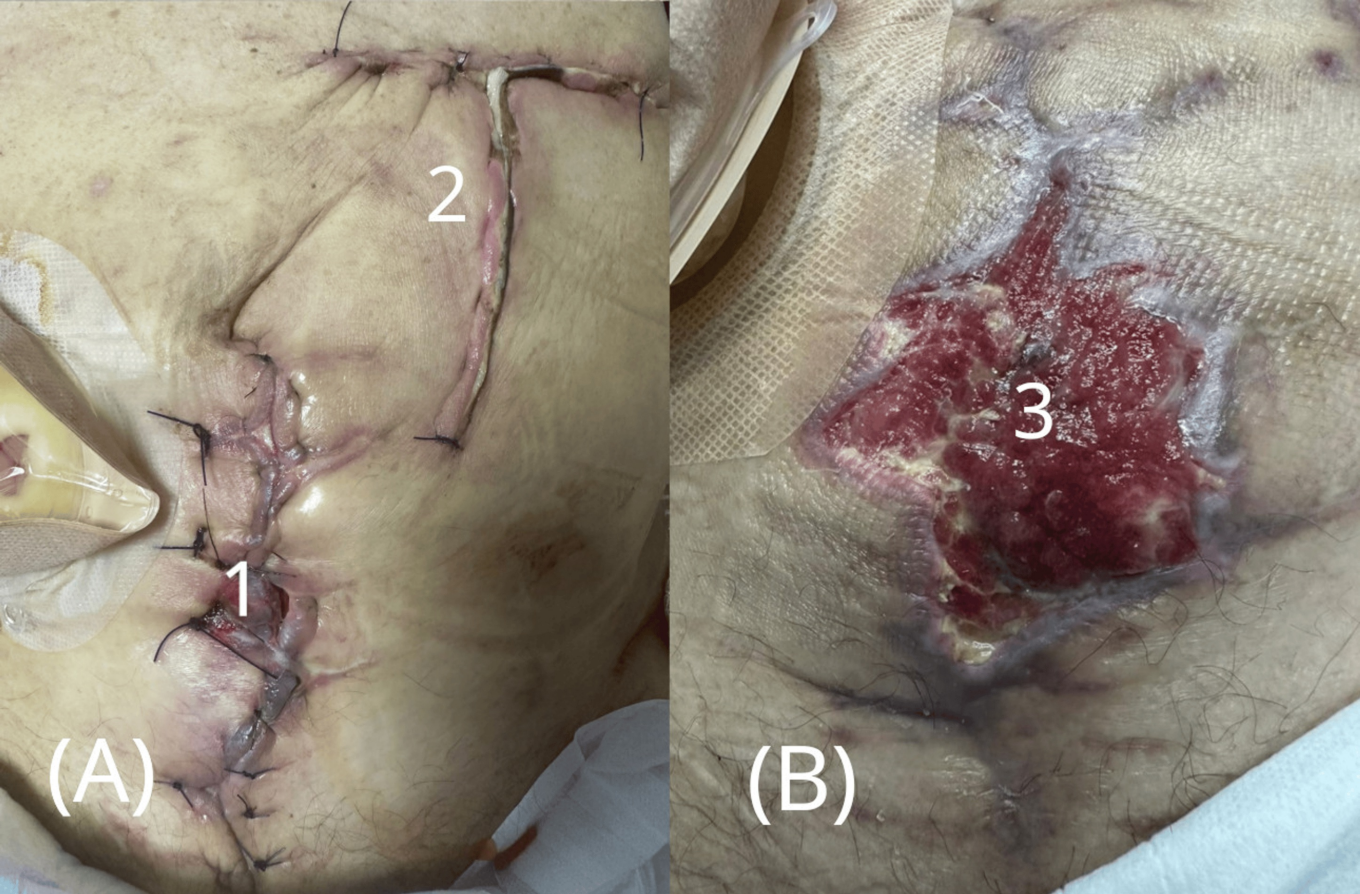
Secondary wound closure and subsequent dehiscence. (A) Attempted wound closure using local skin advancement techniques, with both the original defect and flap site sutured. (B) Re-dehiscence of the wound despite prior closure. 1 - Original abdominal wall defect after closure 2 - Donor site after flap harvest, sutured 3 - New dehiscence site following wound reopening

A third emergency operation was carried out. Necrotic tissue was debrided, and the skin edges were approximated with sutures (Figure 6A). However, wound healing was again unsuccessful, and the sutures loosened, resulting in dehiscence of the skin edges (Figure 6B). NPWT was resumed.

Postoperatively, the patient developed melena and rectal bleeding with associated hemodynamic instability. Treatment included the transfusion of four units of packed red blood cells and two units of fresh-frozen plasma. The symptoms resolved with conservative management.

**Figure 7:**
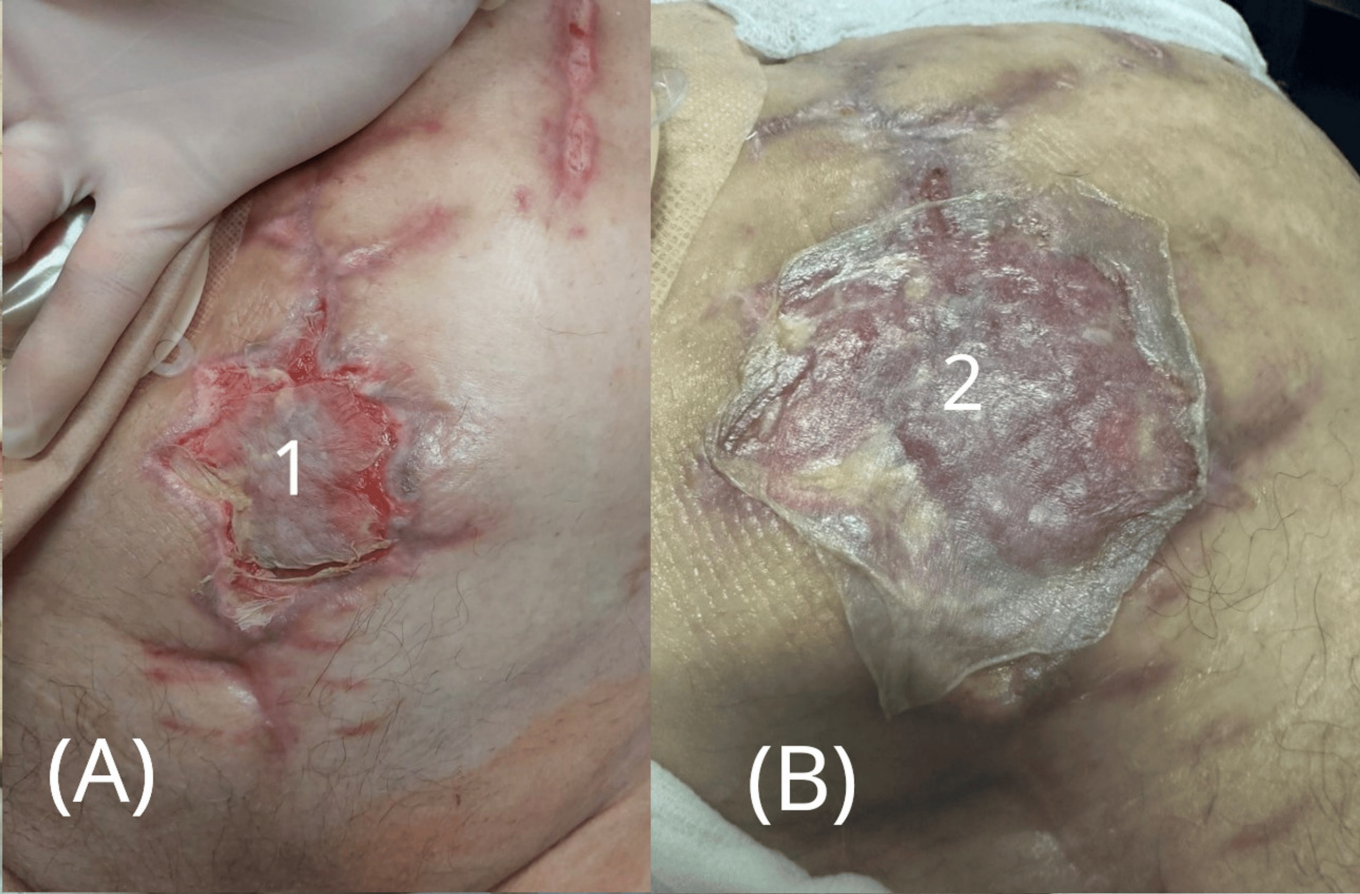
Final wound management with split-thickness skin grafting. (A) Well-granulated wound following completion of negative pressure wound therapy (NPWT), ready for final coverage. (B) Wound appearance after application of a split-thickness skin graft harvested from the thigh. 1 - Healed wound bed after NPWT 2 - Split-thickness skin graft transplanted from the thigh

Over the following weeks, wound healing was monitored with continued use of NPWT. A chronic lower abdominal wall defect persisted (Figure 7A). On January 5, 2024, a split-thickness skin graft harvested from the left thigh was applied under local anesthesia (Figure 7B). The graft was successfully integrated, and the patient was discharged home in stable condition.

**Figure 8:**
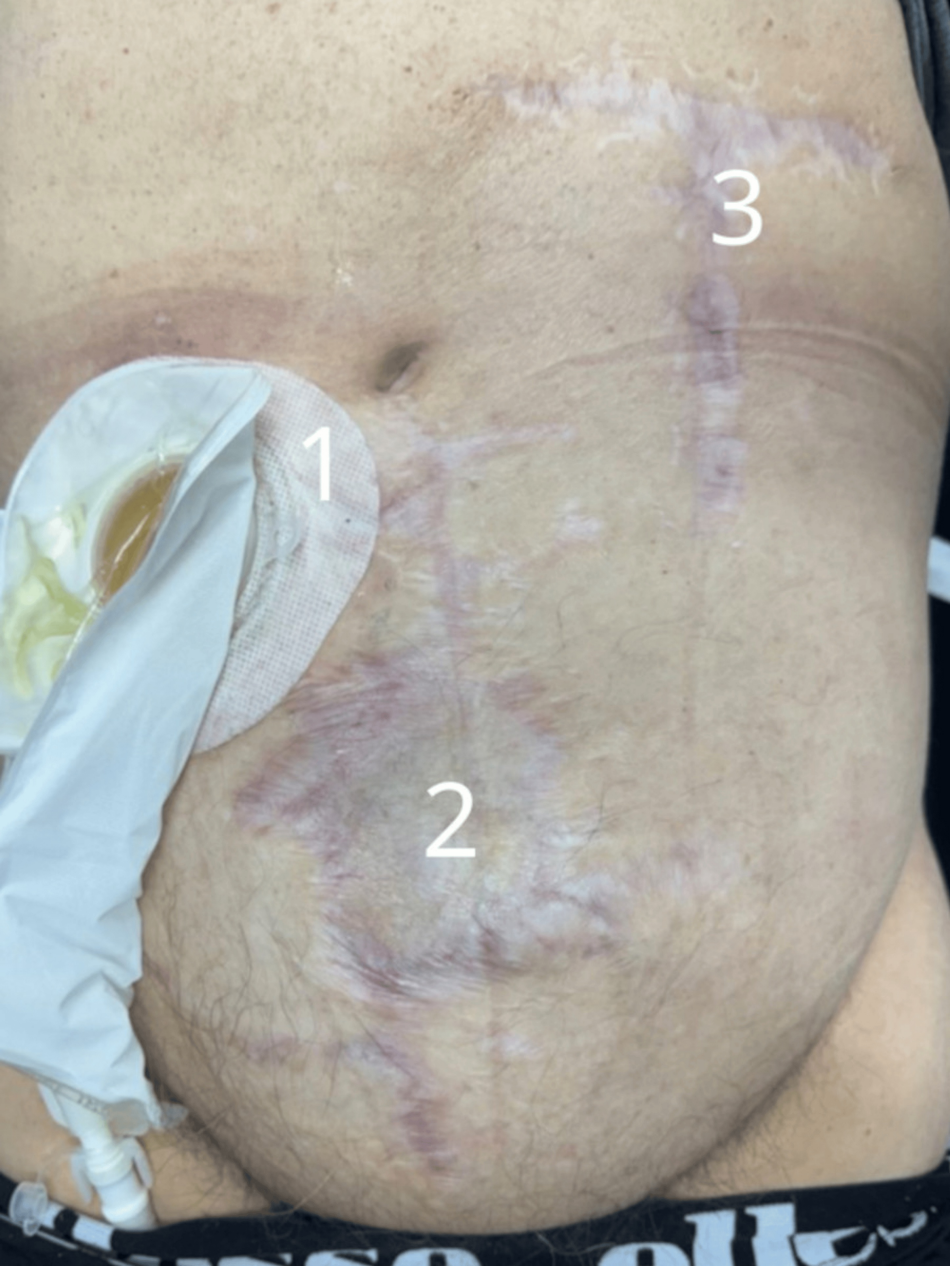
Follow-up examination 10 weeks after final surgery. Clinical image taken at follow-up visit, demonstrating complete wound healing. 1 - Ileal conduit stoma with urostomy bag 2 - Healed abdominal wall at the site of previous eviscerations, with visible scarring 3 - Healed donor site of the pedicled musculocutaneous flap

At follow-up on October 1, 2024, the abdominal wall had fully healed. Both the musculocutaneous flap and the skin graft were well integrated (Figure 8). The patient remained in good overall health, with no recurrence of wound dehiscence, no signs of infection, and satisfactory cosmetic and functional outcomes confirmed on clinical examination and photographic documentation.

## Discussion

This case exemplifies the complex interplay between major oncologic surgery, patient-related risk factors, and postoperative complications. Radical cystoprostatectomy, while curative in localized muscle-invasive bladder cancer, carries substantial perioperative morbidity, with wound dehiscence occurring in approximately 8% of cases following surgery [7]. Evisceration, however, remains a rare complication, accounting for less than 0.5% of cases after laparotomy [3-6].

Abdominal evisceration is associated with multiple risk factors, including age over 65, male sex, impaired wound healing, suboptimal incision or closure technique, hemodynamic instability, increased intra-abdominal pressure (e.g., constipation, cough), emergency surgery, wound or abdominal wall infection, pelvic surgery, ascites, hypoproteinemia, anemia, diabetes, obesity, prior radiotherapy, corticosteroid use, malignancy, and early postoperative physical exertion [8]. The patient’s impaired wound healing was likely multifactorial, with numerous established risk factors including peripheral arterial disease, diabetes, COPD, and a long-standing smoking history, all of which compromised tissue oxygenation and repair. Additionally, a history of ischemic stroke may have further limited his postoperative recovery capacity.

The patient’s compliance was poor. Despite explicit discharge instructions to avoid physical exertion, the patient engaged in household chores, which led to early evisceration. In later stages of hospitalization, he was observed removing surgical drains and urostomy bags, likely increasing wound tension and infection risk. Postoperative delirium and cognitive dysfunction, not uncommon in elderly postoperative patients, likely contributed to these behaviors [9,10].

Initial management with retention sutures and NPWT aligns with standard approaches in the setting of wound dehiscence. However, the size and chronicity of the defect necessitated advanced reconstructive techniques. The use of a pedicled musculocutaneous flap (based on the inferior epigastric artery) is a well-documented method for reconstructing central abdominal wall defects [11]. Despite a technically successful transfer, the skin part of the flap subsequently dehisced. This could reflect either inadequate vascularization, mechanical stress, or both. In such cases, combination therapy with NPWT and staged surgical revision remains the best course of action, as it enables granulation and infection control before final coverage [12]. Ultimately, split-thickness skin grafting provided sufficient coverage and closure of the residual defect. While such grafts do not restore full structural strength, they allow for epithelial closure and reduce the risk of chronic infection or herniation [13].

While the decision involved considerable risk, it was clinically necessary from an oncological standpoint based on the preoperative assessment. The patient was diagnosed with high-grade, muscle-invasive bladder cancer (pT2), and radical cystoprostatectomy remains the standard of care for locally advanced disease, especially after neoadjuvant therapy. Avoiding surgery would likely have led to progression, local symptoms, and worsening quality of life. The decision followed a thorough risk-benefit evaluation, factoring in the absence of distant metastases, the patient’s relatively stable condition, and his informed consent. A multidisciplinary team-including an anesthesiologist, internist, and cardiologist-approved surgical eligibility based on clinical findings. Although the perioperative risk was significant due to multiple comorbidities, this reflects the reality that many uro-oncologic patients undergoing laparotomy are elderly and comorbid, making individualized treatment essential. In this case, a curative approach was pursued, with acceptance of potential postoperative complications.

## Conclusions

This case highlights the need for a holistic and proactive approach to postoperative care in high-risk surgical patients. The decision to operate on a patient with multiple comorbidities must be made with thorough risk evaluation and shared decision-making. Anticipation of impaired wound healing in comorbid patients is crucial; use of advanced wound management strategies, such as prolonged NPWT, and in selected refractory cases, reconstructive options, including musculocutaneous flap, may help reduce cumulative morbidity and surgical burden. Multidisciplinary collaboration, involving urologists, general and plastic surgeons, anesthesiologists, and nursing staff, is key to managing complications and coordinating surgical and wound care. Intensive perioperative education and psychosocial support, including involvement of family, caregivers, or social workers, can make a significant difference in treatment adherence and prevent risky behaviors that compromise recovery.
